# Spectral binning as an approach to post-acquisition processing of high resolution FIE-MS metabolome fingerprinting data

**DOI:** 10.1007/s11306-022-01923-6

**Published:** 2022-08-02

**Authors:** Jasen P. Finch, Thomas Wilson, Laura Lyons, Helen Phillips, Manfred Beckmann, John Draper

**Affiliations:** Institute of Biological, Environmental and Rural Sciences, Aberystwyth University, Aberystwyth, SY23 3DA UK

**Keywords:** Metabolomic fingerprinting, Mass spectrometry, Post-acquisition processing, Software

## Abstract

**Introduction:**

Flow infusion electrospray high resolution mass spectrometry (FIE-HRMS) fingerprinting produces complex, high dimensional data sets which require specialist in-silico software tools to process the data prior to analysis.

**Objectives:**

Present spectral binning as a pragmatic approach to post-acquisition procession of FIE-HRMS metabolome fingerprinting data.

**Methods:**

A spectral binning approach was developed that included the elimination of single scan *m/z* events, the binning of spectra and the averaging of spectra across the infusion profile. The modal accurate *m/z* was then extracted for each bin. This approach was assessed using four different biological matrices and a mix of 31 known chemical standards analysed by FIE-HRMS using an Exactive Orbitrap. Bin purity and centrality metrics were developed to objectively assess the distribution and position of accurate *m/z* within an individual bin respectively.

**Results:**

The optimal spectral binning width was found to be 0.01 amu. 80.8% of the extracted accurate *m/z* matched to predicted ionisation products of the chemical standards mix were found to have an error of below 3 ppm. The open-source R package *binneR* was developed as a user friendly implementation of the approach. This was able to process 100 data files using 4 Central Processing Units (CPU) workers in only 55 seconds with a maximum memory usage of 1.36 GB.

**Conclusion:**

Spectral binning is a fast and robust method for the post-acquisition processing of FIE-HRMS data. The open-source R package *binneR* allows users to efficiently process data from FIE-HRMS experiments with the resources available on a standard desktop computer.

**Supplementary Information:**

The online version contains supplementary material available at 10.1007/s11306-022-01923-6.

## Introduction

Non-targeted metabolome fingerprinting techniques enable the global metabolite analysis of sample extracts (Dunn et al., 2005). Flow infusion electrospray ionisation mass spectrometry (FIE-MS) in particular provides an accessible, robust method for low cost and high throughout metabolite fingerprinting for a wide range of biological matrices (Draper et al., 2013).

Comparing metabolome fingerprints across multiple phenotypes has provided a powerful and unbiased approach for discovering potential molecular perturbations (Southam et al., 2017). It is often applied to perform initial “first pass” analyses, which rapidly provides the user with a comprehensive overview of a broad range of metabolite chemistry in a biological matrix. Potential perturbation relating to differences in phenotype can then be evaluated, and used to generate hypotheses or guide decision making on the next steps of sample analysis or experimentation before the application of more targeted chromatography based techniques (Beckmann et al., 2008). FIE-MS fingerprinting has been used for a wide range of biological applications such as the identification human urinary biomarkers of nutrition and the investigation of plant-fungal pathogen interactions (Lloyd et al., 2013; Parker et al., 2009).

In the last decade, there has been a proliferation in the use of ultra-high resolution (HR) MS instrumentation with Orbitrap mass analysers becoming the standard for HRMS. These mass analysers have provided extraordinary increases in attainable mass resolution, precision and accuracy. But this has also created new challenges for the post-acquisition spectral processing of mass spectra obtained from these instruments with magnitude increases in the volumes of data that can be acquired per sample.

These magnitude increases in data volume require the development of computationally efficient processing routines, especially for processing large sample sets. Spectral artefacts such as Gibbs oscillations, that are common in Fourier Transform based MS, adds further complexity (Marshall & Hendrickson, 2008). Similarly, the alignment of mass spectra between samples can also make processing difficult, especially in analytical runs containing many samples (Draper et al., 2013). Approaches such as that of Smedsgaard and Nielsen (2005) and the *proFIA* R package have already attempted to overcome some of these challenges (Delabrière et al., 2017).

Here, we present a spectral binning based approach as an efficient and pragmatic solution to the post-acquisition processing of FIE-HRMS metabolome fingerprinting data. This is accompanied by the development of the openly available R package *binneR* as a user-friendly implementation of the approach.

## Materials and methods

### Biological and chemical standards mix sample preparation

Samples were used from four example biological matrices. These included leaf tissue from the model grass species *Brachypodium distachyon* (Draper et al., 2001), human urine, human plasma and horse serum. These were prepared and extracted as described in the supplementary materials and methods.

A diverse mixture of chemical standards from a validated assay was prepared by dissolving in a solvent mixture of methanol, water and formic acid (70%:30%:0.1%), each at a concentration of 2mg/ml (Beckmann et al., 2020). This mixture contained a total of 31 standards, for which a list of the names and InChi chemical identifiers can be found in Supplementary Table S1.

### Flow infusion high resolution fingerprinting

Mass spectra were acquired on an Exactive Orbitrap (ThermoFinnigan, San Jose CA) mass spectrometer, which was coupled to an Accela (ThermoFinnigan, San Jose CA) ultra-performance liquid chromatography system. A sample volume of 20 μL was injected and delivered to the electro-spray ionisation (ESI) source via a ‘plug’ flow solvent of pre-mixed HPLC grade MeOH (Fisher Scientific) and ultra-pure H_2_O (18.2 $\Omega$) at a ratio of 7:3. The initial flow rate was 60 μL min^−1^ for 0.4 mins and increased to 200 μL min^−1^ over 0.8 mins. The flow rate was maintained at 200 μL min^−1^ for 0.3 mins then increased to 600 μL min^−1^ over 1.5 mins. Then the flow rate was returned to 60 μL min^−1^ for 0.5 minutes. The total gradient time was 3.5 mins. The capillary temperature and voltage were set at 270 °C and 37.50 kV respectively.

For the *B. distachyon* leaf and human urine samples, mass spectra were acquired using a single scan filter for each of the positive and negative ionisation modes. Positive ions were acquired between 55.000 and 1000.000 *m/z*, and 63.000 and 1000.000 *m/z* for negative ions. Mass spectra for the horse serum, human plasma and standards mix samples were acquired with two scan events between 55–280 *m/z* and 270–1200 *m/z* for each of the positive and negative acquisition modes. For all scan events, the scan rate was 1 Hz with a mass resolution of 100,000. The automatic gain control (AGC) target was 5 × 10^5^ and total ion injection time 250 ms.

Each biological matrix and the chemical standards mix were analysed in separate analytical runs. A total of 10 and two replicate injections were performed for the biological matrices and standards mix respectively. Three blank injections were preformed prior to the analytical replicate injections, with the solvent mixture used in the sample preparation of the particular matrix.

Following data acquisition in profiling mode, raw mass spectra data files (.RAW, ThermoFinnigan) were converted to the universal mass spectrometry open file format, mzML (Martens et al., 2011). Conversion and centroiding were performed using msconvert from Proteowizard (Chambers et al., 2012). All further processing of mzML files was performed using the R Statistical Programming Language version 4.2.1 (R Core Team, 2020).

### Spectral binning and accurate *m/z* assignment

**Fig. 1:**
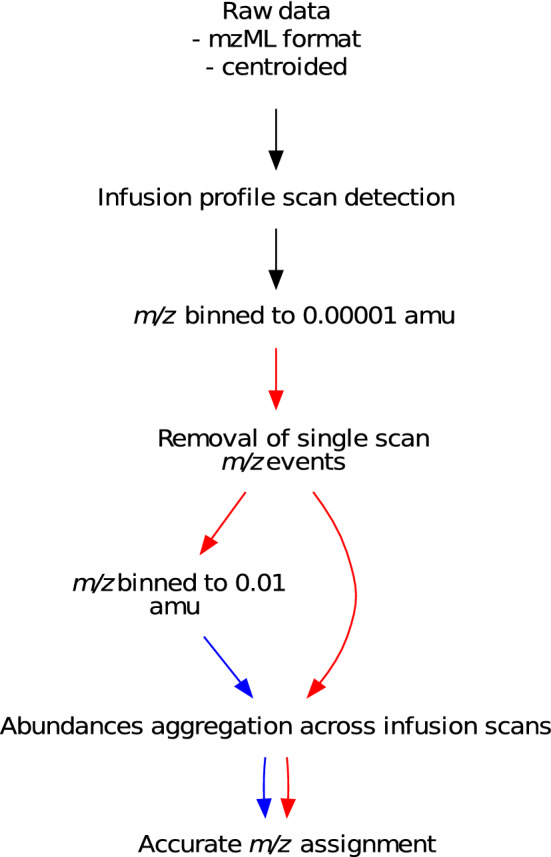
The spectral binning approach for post-acquisition processing of FIE-HRMS metabolomic fingerprinting data. The blue and red arrows denote the actions applied to 0.01 and 0.00001 amu binned data respectively

**Fig. 2:**
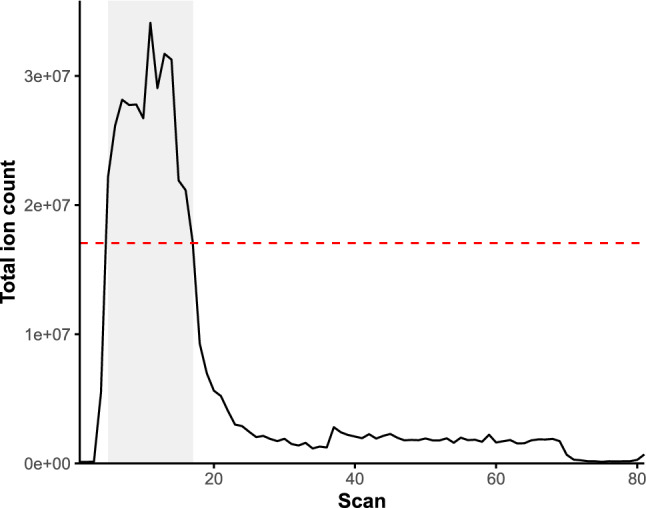
An example FIE-HRMS ion chromatogram. The grey area shows plug flow region that is extracted for spectral binning. The red dashed line shows the 50% level of the maximum ion count, above which scans are selected for processing

An overview of the spectral binning approach used here is shown in Fig. 1. Firstly, for each set of sample types, the scans within the ‘plug’ flow range of the infusion profile were firstly detected by averaging the chromatographic profiles across the scan ranges and ionisation modes of all the replicate injections. The ‘plug’ flow scans were then identified in the average chromatographic profile as those with a total ion count (TIC) greater than 50% of the scan with the highest TIC (Fig. 2).

For each individual sample, the *m/z* were binned to 0.00001 amu within each scan by rounding the *m/z* to five decimal places and sum aggregating the ion abundances within each bin. If multiple scan ranges were present, these were combined, with the average abundances calculated for any *m/z* present across scans of overlapping scan range within an ionisation detection mode. The 0.00001 amu bins were then also binned to 0.01 amu using the same method as for the 0.00001 amu bins. Where necessary, alternative bin widths of 1, 0.1, 0.001 and 0.0001 were also used for comparative purposes. See Section 3.1 for a discussion of the optimal amu bin width. The 0.01 amu bins that contained only a single 0.00001 amu *m/z* that appeared in one single scan in one sample from across the sample set were removed. The abundances of both 0.01 and 0.00001 amu bins were then averaged across the ‘plug’ flow scans and each of the 0.01 amu bins were assigned an accurate *m/z* based on the modal 0.00001 amu *m/z* value within the given 0.01 amu range.

### Bin metrics

Two bin metrics were developed to allow the objective assessment of both the spread and location of *m/z* signals within a given bin window. Bin purity and centrality are defined in the sections below.

#### Purity

The bin purity metric provides a measure of the spread of *m/z* across the *m/z* range of a given bin window. For a given bin containing $n$ mass spectral signals (*m/z*) $m = \{m_1,m_2,\ldots ,m_n\}$ with abundances $a = \{a_1,a_2,\ldots ,a_n\}$, the bin total ion count $t$ can be defined as:

$$\begin{aligned} t = \sum \limits _{i=1}^{n}a_i \end{aligned}$$

The mean *m/z* of the bin:

$$\begin{aligned} {\bar{m}} = \frac{\sum \limits _{i=1}^{n}(m_ia_i)}{t} \end{aligned}$$

The mean absolute *m/z* error for a given bin is given by:

$$\begin{aligned} {\bar{e}} = \frac{\sum \limits _{i=1}^{n}{(a_i|m_i - {\bar{m}}|)}}{t}\end{aligned}$$

Finally, bin purity for a bin of width $w$ (amu) can be defined by:

$$\begin{aligned} p = 1 - \frac{{\bar{e}}}{0.5 \cdot w} \end{aligned}$$

This gives a score between 0 and 1 with higher bin purity denoting a lower spread of *m/z* values within the bin and therefore a lower likelihood of more than one real mass signal being present within the given bin. Examples of high and low purity bins are shown in Supplementary Fig. S1.

#### Centrality

The bin centrality metric provides a measure of the proximity of the mean *m/z* to $k$, the centre of a given bin window. This is given by:

$$\begin{aligned} c = 1 - \frac{|{\bar{m}} - k|}{0.5 \cdot w} \end{aligned}$$

Similarly to bin purity, this provides a score of between 0 and 1 with higher bin centrality denoting a closer proximity of the *m/z* to the centre of the bin. Examples of high and low centrality bins are shown in Supplementary Fig. S2.

### Assignment of measured accurate *m/z* to compounds in the chemical standards mix

Using six common negative ionisation mode adducts ([M − H]^1−^, [M + Cl]^1−^, [M + ^37^Cl]^1−^, [M + K − 2H]^1−^, [2M − H]^1−^, [M − 2H]^2−^) and six common positive ionisation mode adducts ([M + H]^1+^, [M + K]^1+^, [M + ^41^K]^1+^, [M + Na]^1+^, [2M + H]^1+^, [M + 2H]^2+^), possible ionisation products were computed based on the MZedDB ionisation ‘rules’ along with their theoretical *m/z* (Draper et al., 2009). The calculated adduct *m/z* were then matched with a search range of 10 ppm to the accurate *m/z* assigned to the 100% fully occupied 0.01 amu bins of the 10 replicate injections of the standards mix.

### Performance benchmarking of the *binneR* R package

Both processing time and peak random access memory (RAM) usage were tested for the R package *binneR* v2.6.2. These tests were performed on a Dell high performance computing blade with an Intel(R) Xeon(R) Gold 6130 CPU @ 2.10GHz model processor with 128 CPUs, 504GB of RAM and Ubuntu 20.04.4 LTS operating system.

Increments of 1, 10, 100, and 1000 raw data files were used from the *B. distachyon* leaf sample matrix. The data files were duplicated 10 and 100 times for the 100 and 1000 file tests respectively. Processing time and peak memory usage tests were performed separately for each combination of the numbers of files and the numbers of CPU workers in increments of 1, 4, 16 and 64. The R package *rbenchmark* v1.0.0 was used for measuring processing time with a single replication of each file number-CPU workers combination. The R package *profvis* v0.3.7 was used to profile RAM usage with usage sampled at 0.01 second intervals for all combinations except for those with 1 CPU worker and greater than 10 data files that were sampled at 0.5 second intervals. The peak RAM usage was taken as the maximum RAM usage observed during each test.

## Results and discussion

### Optimal bin width for spectral binning

The optimal width with which to spectral bin FIE-HRMS fingerprinting data is a compromise between the retention of resolution whilst minimising the impacts of instrumental or processing artefacts.

As the amu width of spectral binning decreased, there was an exponential increase in the number of spectral features observed along with an increase in the proportion of missing values across all the example biological matrices (Supplementary Tables S2 and S3). This was a result of the measured deviation in *m/z* signals both between each scan of a sample and between samples. The peak at 133.01416 *m/z* from the *B. distachyon* leaf matrix was found to deviate by a range of up to 0.000244 amu between the infusion scans of a single injection and 0.0000777 amu across the 10 injections (Supplementary Fig. S3). These deviations are due changes in parameters such as temperature and space charge compensation during the Fourier transformation, which is calculated on a scan by scan basis (Hu et al., 2005). This means that *m/z* have the potential to freely shift between bins at widths of 0.0001 or less and so contribute to the high proportions of missing data. Due to this, the use of bin widths of 0.0001 amu and below would be unsuitable for spectral binning.

A higher bin width increases the likelihood that one bin will contain peaks from multiple compounds. This can have implication for downstream data analysis as feature trends become convoluted and more difficult to interpret in the context of the biological question. It also makes putative annotation of features more difficult as the correlations between bins become less reflective of the underlying isotopic, adduct and biochemical relationships. This was a major limitation of former nominal mass fingerprinting techniques using low resolution mass analysers (Beckmann et al., 2008)

**Fig. 3:**
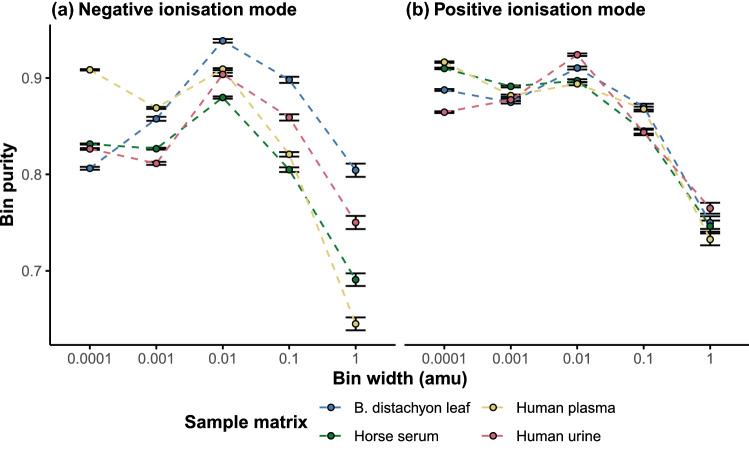
The average bin purity at incremental binning width (amu) across the example biological sample matrices for each ionisation mode. Bin purity was averaged across all bins from the 10 technical injections of each matrix. The error bars show the standard error of the mean

The bin purity metric provides a means by which potential co-occurrence can be compared between different binning widths. Bin purity was found to be variable between the example biological matrices however it was consistently highest on average at 0.01 amu (Fig. 3). At the 0.01 amu binning width, there is the lowest spread of *m/z* relative to the width of the bin.

Imposing discrete bins of any amu width across a mass range where mass deviation can occur could lead to the splitting of peaks between bins where peaks occurs near bin boundaries. At smaller amu bin widths, the chance of this occurring increases as more bin boundaries are present. The result of a split peak would be two negatively correlated, adjacent bins obtained for what in reality is a single peak where the peak falling into one or other of the bins for a given scan (Supplementary Fig. S4). The proliferation of these artificial features could lead to over optimism during downstream modelling where variance is utilised that is unrelated to the biological problem (Enot et al., 2008; Worley and Powers, 2013).

It was found that of the predicted ionisation products for the standards mix, 33 of these were matched to multiple adjacent bins when the data were binned using a width of 0.001 amu, compared to just one ionisation product at 0.01 amu. This shows that there is a magnitude increase in the potential for peaks to be split between bins as binning width decreases. It is likely that this could be further increased in more complex matrices such as those of biological samples that contain hundreds of compounds.

Based on the factors discussed above, the application of the spectral binning approach using a bin width of 0.01 amu provides the best compromise between retaining resolution, while reducing the impacts of missing data and keeping processing artefacts to a minimum.

### Extracted *m/z* accuracy

It is a requisite that the accurate *m/z* assigned to the 0.01 width amu bins using this approach are able to provide sufficient accuracy to enable the putative assignment of molecular formulas and metabolite annotations.

**Table 1:** Extracted accurate *m/z* base peaks for 0.01 amu bins detected for compounds in the mix of chemical standards

Compound	Molecular formula	Adduct	Theoretical m/z	Measured m/z	Abundance	Purity	Centrality	$\Delta$ ppm
Carnitine	C$_{7}$H$_{15}$NO$_{3}$	[M + H]$^{1+}$	162.1125	162.1121	1,350,916	0.996	0.5720	2.040
Creatinine	C$_{4}$H$_{7}$N$_{3}$O	[M + H]$^{1+}$	114.0662	114.0659	1,053,376	0.995	0.1930	2.280
Hydroxyproline betaine	C$_{7}$H$_{13}$NO$_{3}$	[M + H]$^{1+}$	160.0968	160.0966	1,698,621	0.994	0.3060	1.620
Indoxyl Sulfate	C$_{8}$H$_{7}$NO$_{4}$S	[M − H]$^{1-}$	212.0023	212.0022	555,858	0.995	0.5610	0.613
N-Methyl histidine	C$_{7}$H$_{11}$N$_{3}$O$_{2}$	[M + H]$^{1+}$	170.0924	170.0920	464,555	0.998	0.5900	2.180
Nicotine	C$_{10}$H$_{14}$N$_{2}$	[M+H]$^{1+}$	163.1230	163.1225	1,715,843	0.870	0.5480	2.700
p-Cresol sulfate	C$_{7}$H$_{8}$O$_{4}$S	[M − H]$^{1-}$	187.0070	187.0069	2,059,838	0.996	0.3870	0.749
Proline betaine	C$_{7}$H$_{13}$NO$_{2}$	[M + H]$^{1+}$	144.1019	144.1016	1,681,100	0.998	0.6740	2.010
Trans-3’-Hydroxycotinine	C$_{10}$H$_{12}$N$_{2}$O$_{2}$	[M + H]$^{1+}$	193.0972	193.0968	835,054	0.997	0.3550	2.020
Trigonelline	C$_{7}$H$_{7}$NO$_{2}$	[M + H]$^{1+}$	138.0550	138.0547	918,751	0.999	0.0551	1.740

The top ten most abundant base peaks are shown. For a full list of detected peaks see Supplementary Table S4

The accuracy of some of the extracted accurate *m/z* signals from the chemical standards mix are shown in Table 1. A full list of the matched accurate *m/z* can be found in Supplementary Table S4. Out of a total of 347 predicted ionisation products based on the selected common adducts, 151 (43.5%) were matched to accurate *m/z* that had been assigned to 0.01 amu bins. These matches had an average ppm error of 2.35 (SE = ± 0.167) with 122 (80.8%) having an error below 3 ppm. This level of accuracy obtained across the majority of detected features is sufficient to allow for the assignment of putative molecular formulas or metabolite annotations (Kind & Fiehn, 2006)

**Fig. 4:**
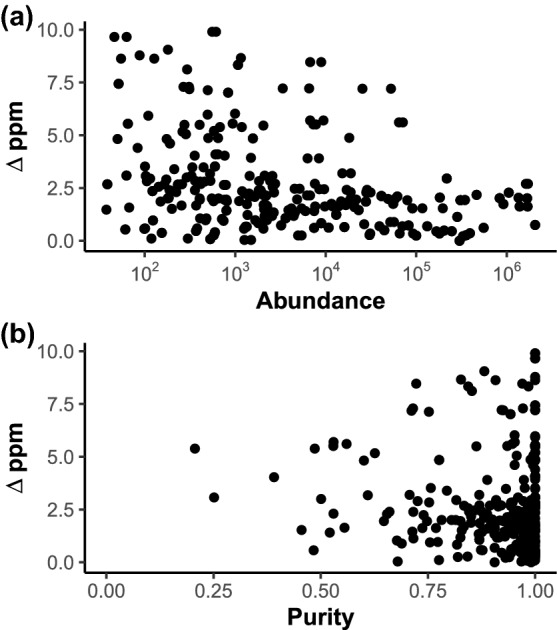
Error of accurate *m/z* matched to the predicted ionisation products in the standards mix plotted against **a** their detected abundance and **b** the calculated 0.01 amu bin purity

As shown in Fig. 4, high ppm error was found to be more associated with low abundance as opposed to low purity. This suggests that low accuracy was as a result of instrumental error as opposed to being introduced during processing by the spectral binning approach (Makarov et al., 2006).

Of the 195 predicted ionisation products whose accurate *m/z* were unmatched, 26 (13.3%) had 0.01 amu bins that were present in the data set. These present, but unmatched, bins had a high average purity of 0.946 (SE = ± 0.0174) suggesting that these bins were not matched because the predicted ionisation products were not detected. This is opposed to their co-occurrence in a bin with higher abundance *m/z* of alternative ionisation products whose accurate *m/z* were instead selected.

### *binneR*: An R package for spectral binning of FIE-HRMS data

The R package *binneR* was developed as an implementation of this spectral binning approach and is available to install from GitHub (https://aberhrml.github.io/binneR/). A usage tutorial is provided within the package for new users.

The package provides utility for the automatic detection of all necessary parameters such as infusion scan detection and scan filters. Parallel processing uses the R package *future*, providing the user with a wide choice of processing strategies (Bengtsson, 2020). Data files should be provided in common mass spectrometry data formats such as those supported by the mzR package available from Bioconductor (https://github.com/sneumann/mzR/) including mzML and mzXML (Chambers et al., 2012; Martens et al., 2011; Pedrioli et al., 2004). There is an integrated workflow for the spectral binning approach described here including 0.01 amu binning, accurate *m/z* retrieval and bin metric calculation. Also included is functionality for visualising ion chromatograms of infusion profiles and spectral bins.

To show the performance of the package for processing FIE-HRMS data, both the processing time and peak memory usage were measured across a range of data file numbers and CPU workers (Supplementary Fig. S5). It was found that processing 100 files using 4 CPU workers took only 55 seconds and had a peak memory usage of 1.36GB. These required resources show that processing hundreds of data files using this implementation is within the capabilities of a standard modern desktop computer. It should also be noted that there was little gain in processing time using 16 or more CPU workers for 1000 files.

### The spectral binning approach

Metabolome fingerprinting is not an ultimately comprehensive method for which metabolome profiling techniques utilising chromatographic separation are available and more suited (Theodoridis et al., 2012). The basis of this spectral binning approach is for first pass analysis where sample throughput, low cost and efficiency, both in time and computational resources are the most important factors. It can be seen as a pseudo-hierarchical approach were some resolution is conceded for simplicity and efficiency. However, it is an attempt to ensure that the post-acquisition processing step is not the inhibitory factor or bottle neck in biological applications where many thousands of samples may be available such as epidemiological or population studies (Watrous et al., 2017).

Binning, used as a form of quantization, is commonly applied in post-acquisition processing approaches for Nuclear Magnetic Resonance (NMR) spectroscopy data (Åberg et al., 2009). Where adaptive binning is often preferred for NMR, this is made challenging in Fourier transform mass spectrometry due to the deviations in measured *m/z* and the presence of spectral artefacts such as Gibbs occilations (Marshall & Hendrickson, 2008). Adaptive binning methods can also significantly increase the computational demands and can require manual intervention to ensure that the bin boundaries have been appropriately applied (Anderson et al., 2011).

The removal of single scan *m/z* events during spectral binning (Fig. 1) enables the reduction of random instrumental noise and can greatly reduce the proportion of missing data in an intensity matrix. Removal of these *m/z* reduced the percentage of missing data in the *B. distachyon* matrix by 21.8% and 25.4% in positive and negative ionisation modes respectively. This reduction diminishes as sample numbers increase due to the greater chance that bins will be multiply occupied.

The proposed bin metrics, along with the visualisation of bin spectra, can be used by the investigator as a means to objectively assess the robustness of individual bins where necessary after processing. Spectral bins found to be explanatory for a given biological question by downstream data analyses and suspected to potentially contain *m/z* from multiple compounds, could be investigated further by applying a peak detection routine such as the continuous wavelet transform (Zheng et al., 2016). The relative intensities can then be compared to potentially identify the *m/z* responsible for the explanatory bin.

An alternative approach for processing FIE-HRMS data by the *proFIA* R package attempts to detect peaks in the chromatographic dimension using a peak picking approach of the infusion profile as opposed to the spectral dimension (Delabrière et al., 2017). This method ensures the detection of the most robust peaks; however, the performance of this approach and peak picking approaches, in general, is that they could be susceptible to information loss. Peaks with poor shape, as a result of short infusion profiles or low abundance, could be missed or incorrectly picked by these routines. The vast majority of *m/z* signals found within FIE-HRMS spectra are of low abundance and close to the baseline. However, these features can often still provide useful information for downstream data mining and it is important that this information is retained.

The poor performance of a peak picking routine could not only affect the precision of an extracted m/z but also the precision of the extracted abundance. Comparatively, a sum aggregated spectral bin with a low bin purity, will only affect the precision of the assigned accurate mass of the bin. While the poor performance of both approaches would affect the potential to assign molecular formulas to accurate *m/z*, the poor performance of peak picking approaches would be more detrimental to downstream data mining.

These peak picking approaches would also be unsuitable for processing direct infusion mass spectrometry (DIMS) data where the ion current is held stable for a period of time (Southam et al., 2017). This would also yield peak shapes unsuitable for peak pick routines; however, the spectral binning approach would still be suitable for processing this data.

Spectral binning is also comparatively faster than the *proFIA* approach, with processing taking approximately 1 second per file compared to the reported speed of approximately less than 15 seconds (Delabrière et al., 2017). The slower speed of *proFIA* is likely due to the complexity of the extra computational steps involved in the approach.

Due to the simplicity of the spectral binning approach, a number of quality assurance steps should be taken to ensure that robust features can be selected during pre-treatment routines prior to downstream data mining. This includes the block randomisation of sample classes across sample runs, which should be mandatory to avoid the introduction systematic error (Beckmann et al., 2008). Accompanying this should be use of quality control (QC) samples, samples that are representative in composition of all the samples to be analysed (Broadhurst et al., 2018). The QC samples should be injected at the start of the run and between the randomised class blocks. This allows bins to be filtered based on their relative standard deviation (RSD) across the sample run and only robustly measured bins with RSD values below a threshold value retained for further analysis. Thresholds of below 20% and 30% have been recommended for LC-MS and GC-MS based investigations; however a higher threshold of 50% would be more suitable for FIE-HRMS, given the more complex ionisation environment during sample infusion (Dunn et al., 2012).

Bins containing a high percentage of missing values or low occupancy should also be removed as these can represent poorly detected and noisy features. A recommended strategy is that for a bin to be retained, it should be occupied above a threshold of 66% in at least one of the sample classes (Southam et al., 2017). Imputation on the remaining missing values can then be performed using approaches such as random forest or k-nearest neighbour imputation (Hrydziuszko & Viant, 2012; Kokla et al., 2019)

## Conclusions

The spectral binning approach presented here provides an efficient and pragmatic approach for for post-acquisition processing of FIE-HRMS metabolome fingerprinting data. The optimal *m/z* binning width was found to be 0.01 amu where an initial concession of resolution for processing efficiency is offset by the per bin extraction of modal accurate *m/z*. For the mix of known chemical standards, it was shown that the majority of extracted accurate *m/z* could be matched to the predicted ionisation products with an accuracy below 3 ppm. The purity and centrality metrics also provided an objective means for investigators to assess the robustness of individual bins.

The development the open-source R package *binneR* provides an efficient implementation of the approach that makes the processing of hundreds of FIE-HRMS data files possible on an ordinary desktop PC.

## Supplementary Information

Below is the link to the electronic supplementary material.

Supplementary file1 (PDF 92 kb)

Supplementary file2 (PDF 118 kb)

Supplementary file3 (PDF 33 kb)

Supplementary file4 (CSV 3 kb)

Supplementary file5 (CSV 19 kb)

## Data Availability

The metabolomics data reported in this paper are available via the EMBL-EBI MetaboLights database with the study identifier MTBLS4288 (https://www.ebi.ac.uk/metabolights/MTBLS4288). The R code used to analyse the data and generate this manuscript is available via GitHub at https://github.com/jasenfinch/Spectral_binning_as_an_approach_to_post-acquisition_processing_of_FIE-HRMS_data.

## Code availability

The R package *binneR* is available for download from GitHub at https://aberhrml.github.io/binneR/.
