# Supplementary material for: Spectral binning as an approach to post-acquisition processing of high resolution FIE-MS metabolome fingerprinting data
Source: Metabolomics. 2022 Aug 2;18(8):64. doi: 10.1007/s11306-022-01923-6 (PMC9345815; doi:10.1007/s11306-022-01923-6)
Supplement: Supplementary file 1 — Supplementary file1 (PDF 92 kb) [file 11306_2022_1923_MOESM1_ESM.pdf]

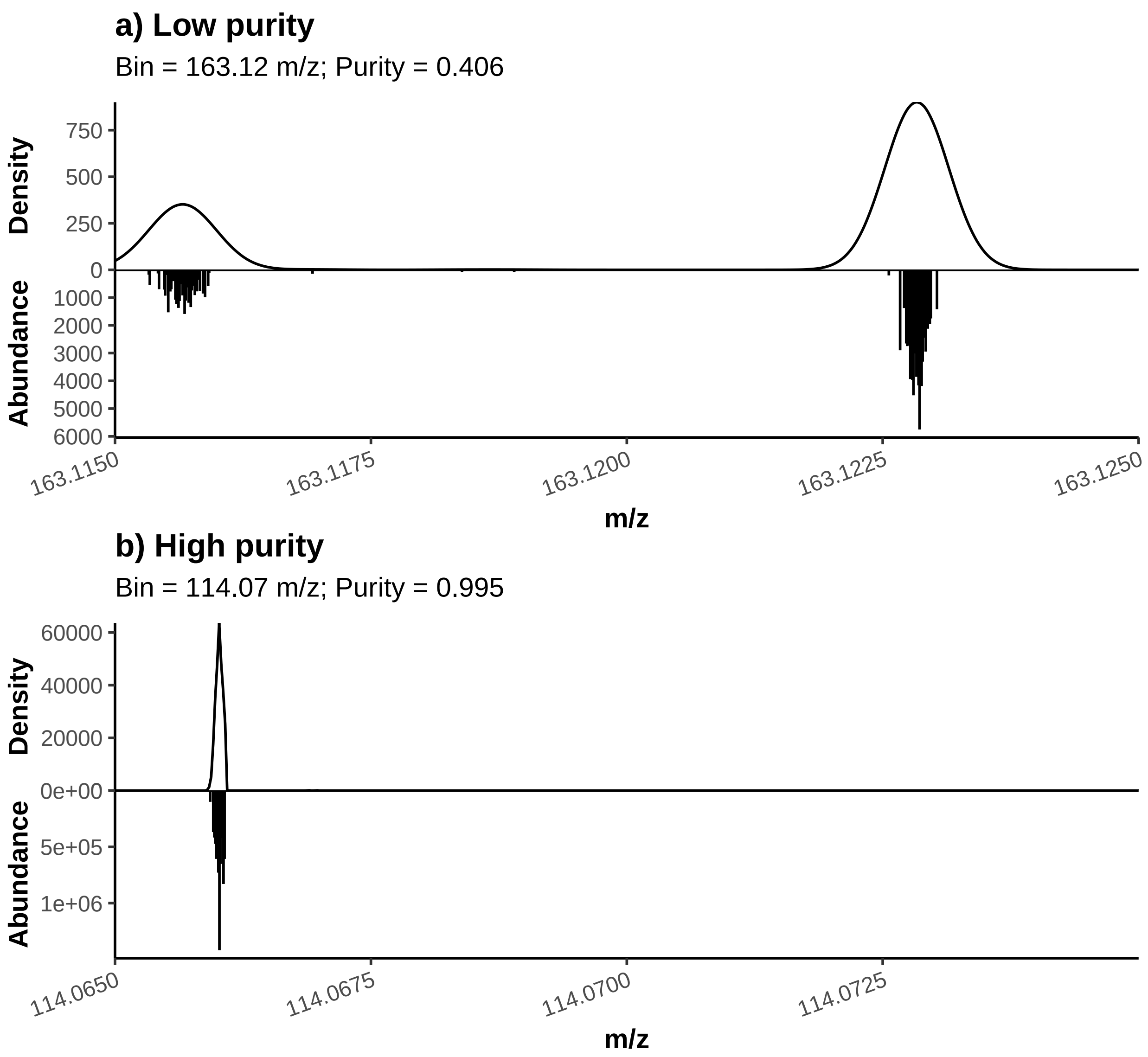

Figure S1: Examples of low and high purity 0.01 amu spectral bins. Both bins were taken from the human urine positive ionisation mode example matrix. The upper half of the graph shows the kernal density estimate distribution of the abundance spectrum below.

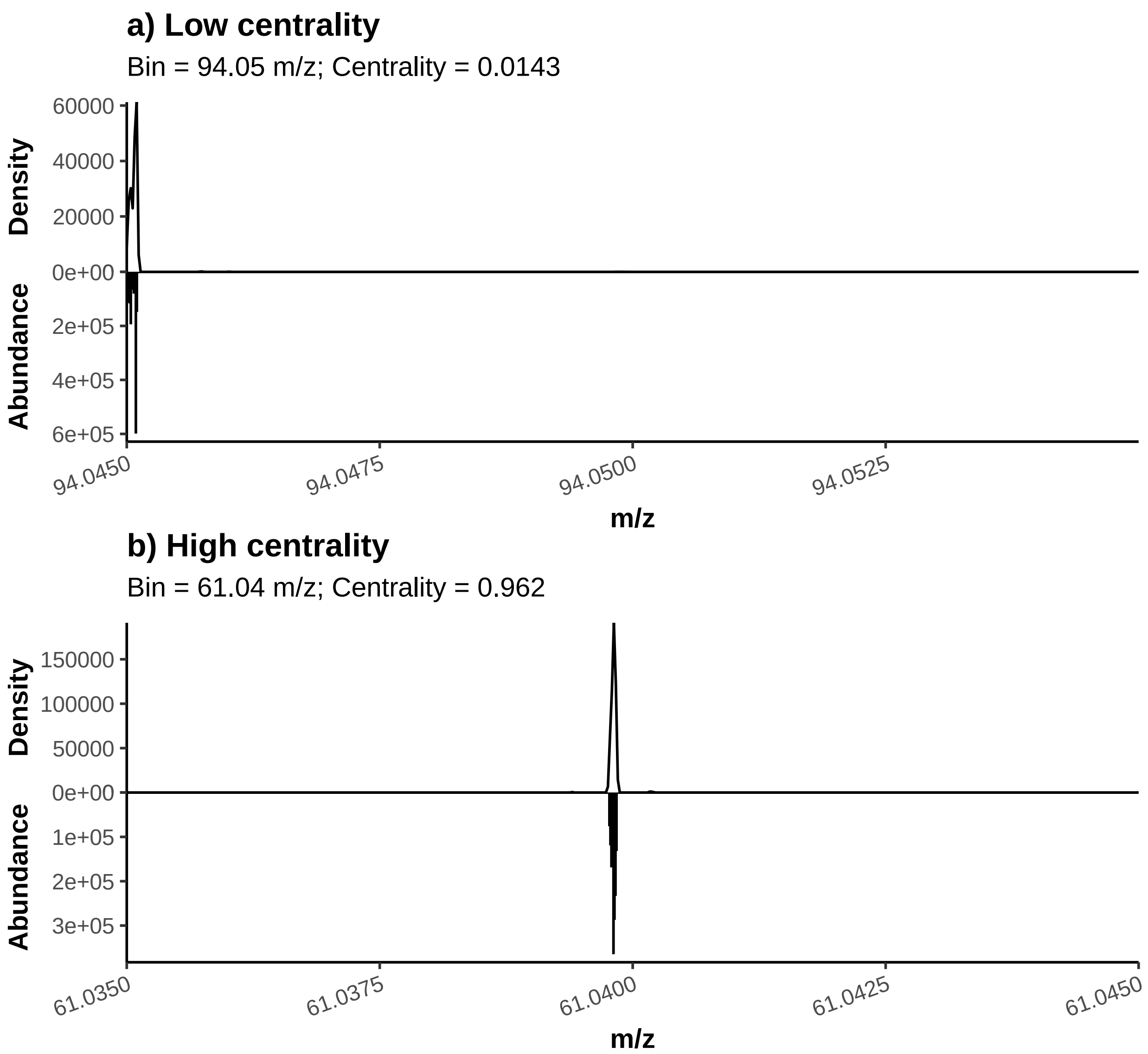

Figure S2: Examples of low and high centrality, 0.01 amu spectral bins. a) Bin 94.05 *m/z* was taken from the *B. distachyon* leaf positive ionisation mode example matrix. b) Bin 61.04 *m/z* was taken from the human urine positive ionisation mode example matrix.

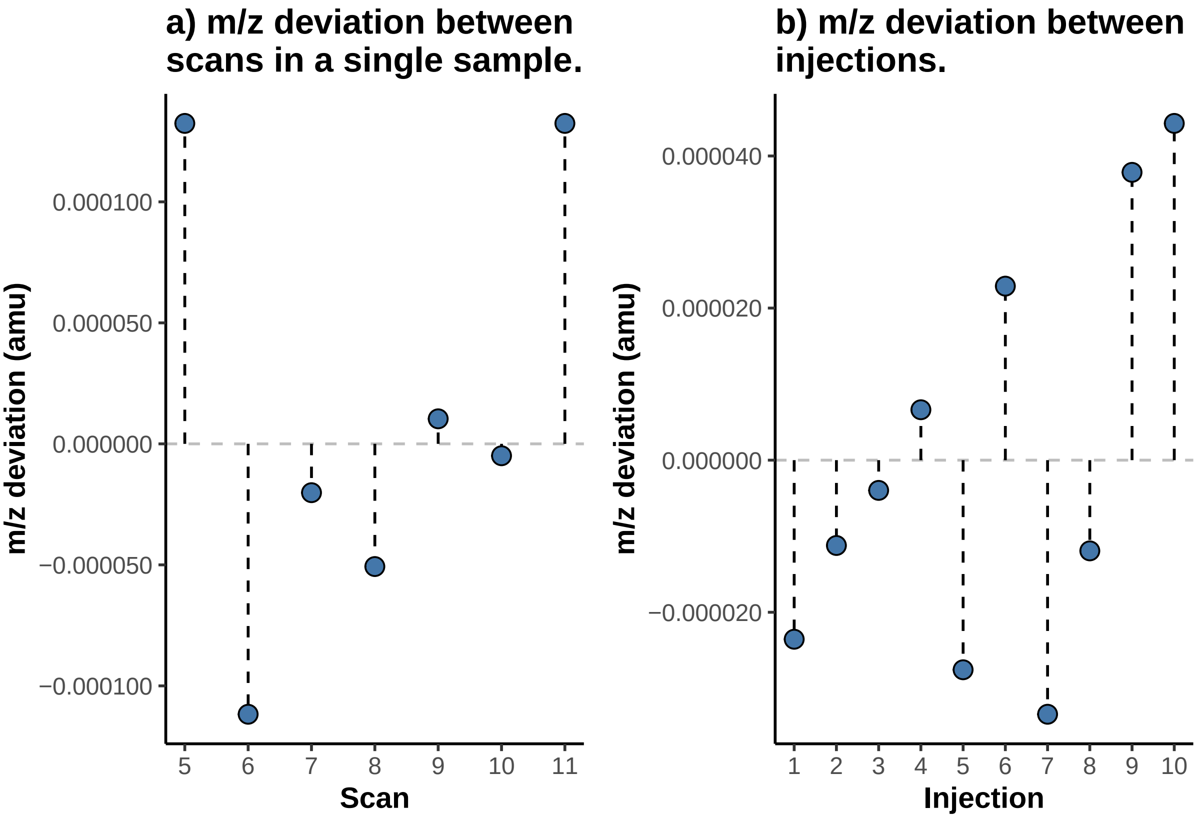

Figure S3: Deviations in *m/z* for an example peak between the scans of a single sample and across replicate injections. *m/z* deviations taken from the base peak at 133.01416 *m/z* in 10 replicate injections of the *B. distachyon* leaf negative ionisation mode example matrix. The *m/z* deviations between the scans of the single sample were taken from the detected 'plug' flow of the first replicate

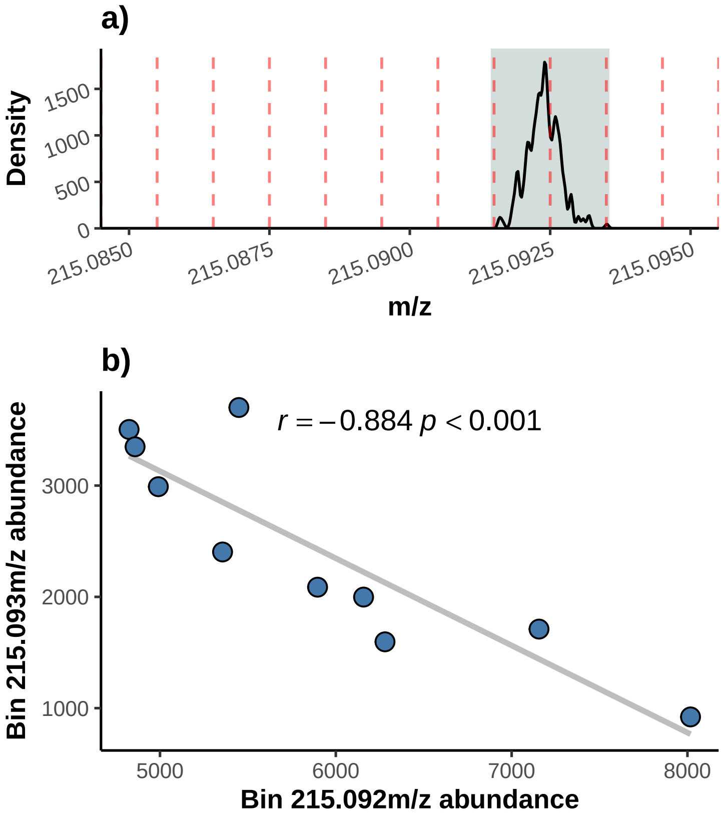

Figure S4: An example of peak "splitting" at 0.001 amu bin width. a) A kernal density distribution plot of the negative ionisation mode bin at 215.09 m/z, averaged across the 10 replicate injections of the example urine sample matrix. The red lines denote the 0.001 amu bin width boundaries. The grey shaded areas denote the 0.001 amu adjacent bins that are plotted in (b). b) A scatter plot of the abundances for the adjacent 0.001 amu bins 215.092 and 215.093 in negative ionisation mode for the 10 technical injections of the example urine sample matrix. The pearsons correlation coefficient between the bins is shown along with its p-value.

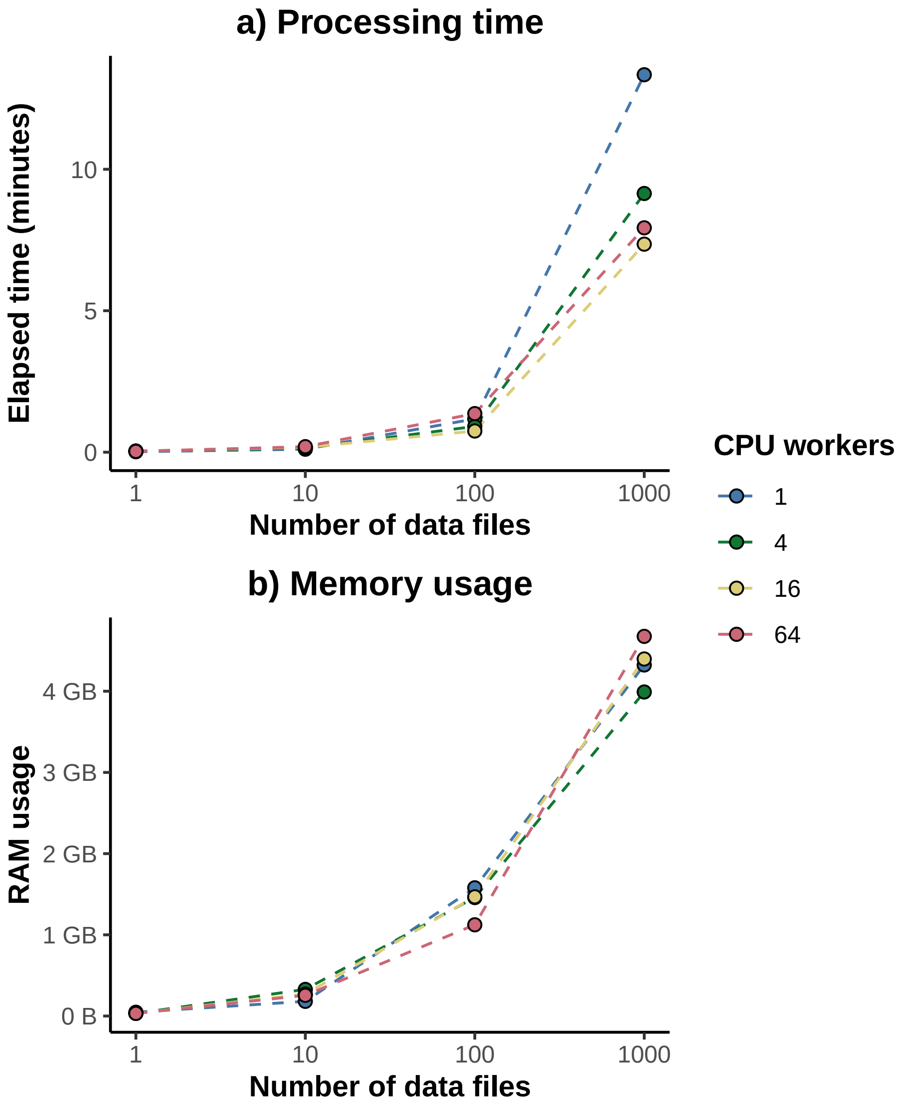

Figure S5: Performance benchmarking results of processing time and peak memory usage for the binneR R package. The raw data files from the 10 replicate injections of the *B. distachyon* leaf sample matrix were used. The data files were duplicated 10 and 100 times for the 100 and 1000 file tests respectively.
