# Supplementary material for: Spectral binning as an approach to post-acquisition processing of high resolution FIE-MS metabolome fingerprinting data
Source: Metabolomics. 2022 Aug 2;18(8):64. doi: 10.1007/s11306-022-01923-6 (PMC9345815; doi:10.1007/s11306-022-01923-6)
Supplement: Supplementary file 2 — Supplementary file2 (PDF 118 kb) [file 11306_2022_1923_MOESM2_ESM.pdf]

Supplementary materials and methods

Sample preparation and extraction protocols

Plant tissue collection and extraction

Plants of *Brachypodium distachyon* ecotype ABR1 were grown for 21 days in environmentally controlled growth rooms (Polysec, R. J. Hicks Refrigeration, Aberystwyth, UK) at 23 °C under a 16 hour light period. A total of 7 *B. distachyon* plants were harvested by detaching the 2nd fully developed leaf from the base of the plant and disecting the middle 4 cm segment. This was then placed in a 2 mL Eppendor tube containing a 4mm steel ball bearing and snap frozen in liquid nitrogen. Samples were stored at -80 °C until extraction. All harvesting was conducted in the growth room to avoid environmental fluctuations.

The frozen samples were milled using a Retsch mm 301 Mixer Mill at 30 Hz for 30 seconds then placed on crushed ice. Pre-chilled extraction solvent ($CHCl_3$:MeOH:H_2O; 1:2.5:1; v:v:v) was immediately added using 700 μL/leaf and suspended by vortexing (Scientific Industries Vortex Genie-2). Samples were then placed on a orbital shaker (FATSN002, Favorgen Biotech Corp) for 20 minutes at 1,400 rpm and a temperature of 4 °C. After shaking, samples were centrifuged (EBA 12R, Hettich) at 13,000 rpm for 6 minutes at 0 °C. The supernatant was then transferred and combined in a 5 mL Eppendorf tube and the pellet discarded giving a total extract volume of 4.9 mL. The sample was stored at -80 °C until MS analysis.

Human urine collection and extraction

Human urine was collected from 12 healthy individuals. Each individual collected approximately 40 mL of mid stream urine 2-3 hours post evening meal. All urine was pooled and throughly mixed. Pooled was urine was dispensed into 500 μL aliquouts and stored at -80 °C. 500 μL of pooled urine was thawed to room temperature and transferred to a clean 2 mL Eppendorf tube. 500 μL of pre-chilled MeOH (Extraction Grade, Fisher Scientific) was added and the sample vortexed. Samples were then placed on an orbtial shaker (FATSM002, Favorgen Biotech Corp) for 20 minutes at 1,400 rpm and 4 °C in the dark. The samples were centrifuged (EBA 12 R, Hettich) at 13,000 rpm for 5 minutes at 4 °C and 20 μL of the supernatunt transfered to clean glass HPLC vial, containing a 200 μL flat bottom micro insert (Chromacol) and diluted with 80 μL of H_2O:MeOH (3:7) directly in the vial.

Human plasma and horse serum extraction

Pooled, mixed gender, lithium heparin treated, human plasma (Seralab) and horse serum (Seralab) were extracted using the following method. 200 uL of raw plasma or serum was transferred to a 2 mL Eppendorf tube, containing 1 micro spoon of glass beads. 1520 uL of pre-chilled methanol/chloroform (4:1, v/v) was added and the sample vortex. The sample was shaken for 15 min at 4°C and then stored at -20°C for 20min. The sample was then centrifuged at 14,000 rpm and 4°C for 5min. 20uL of the supernatant was transferred to a clean glass vial and diluted with 80 uL of 70:30 (methanol/water).
