# Supplementary material for: Spectral binning as an approach to post-acquisition processing of high resolution FIE-MS metabolome fingerprinting data
Source: Metabolomics. 2022 Aug 2;18(8):64. doi: 10.1007/s11306-022-01923-6 (PMC9345815; doi:10.1007/s11306-022-01923-6)
Supplement: Supplementary file 3 — Supplementary file3 (PDF 33 kb) [file 11306_2022_1923_MOESM3_ESM.pdf]

Table S2: Total numbers of bins at incremental amu bin width detected across the 10 replicate injections of each of the example biological sample matrices for each ionisation modes.

		Total number of bins	
Bin width (amu)	Biological matrix	Negative mode	Positive mode
0.00001	Brachypodium leaf	18547	13452
	Horse serum	60076	19768
	Human plasma	22973	17960
	Human urine	26220	25159
0.00010	Brachypodium leaf	12866	13186
	Horse serum	66852	16829
	Human plasma	23384	15702
	Human urine	30772	19067
0.00100	Brachypodium leaf	3960	7515
	Horse serum	24644	10525
	Human plasma	15096	10184
	Human urine	10236	8737
0.01000	Brachypodium leaf	1911	4504
	Horse serum	13038	8193
	Human plasma	10320	7225
	Human urine	4123	5214
0.10000	Brachypodium leaf	1479	2448
	Horse serum	5540	4265
	Human plasma	5959	4020
	Human urine	2103	3033
1.00000	Brachypodium leaf	702	826
	Horse serum	1142	1133
	Human plasma	1143	1127
	Human urine	821	916

Table S3: The percentage of missing data detected at incremental amu bin width across the 10 replicate injections of each of the example biological sample matrices for each ionisation mode.

		Missing data (%)	
Bin width (amu)	Biological matrix	Negative mode	Positive mode
0.00001	Brachypodium leaf	76.30	74.40
	Horse serum	78.00	74.50
	Human plasma	75.50	74.70
	Human urine	72.30	71.90
0.00010	Brachypodium leaf	56.60	66.80
	Horse serum	64.70	67.40
	Human plasma	70.00	68.30
	Human urine	67.90	62.90
0.00100	Brachypodium leaf	41.20	52.40
	Horse serum	47.40	53.70
	Human plasma	54.90	54.70
	Human urine	41.80	48.70
0.01000	Brachypodium leaf	39.60	44.60
	Horse serum	43.90	48.80
	Human plasma	46.20	48.10
	Human urine	37.80	43.50
0.10000	Brachypodium leaf	46.10	36.40
	Horse serum	30.60	38.60
	Human plasma	35.80	36.00
	Human urine	41.20	43.30
1.00000	Brachypodium leaf	29.60	23.80
	Horse serum	5.94	9.96
	Human plasma	1.66	7.51
	Human urine	33.70	23.40
